# An assessment of the HIV/TB knowledge and skills of home-based carers working in the North West province in South Africa: a cross-sectional study

**DOI:** 10.1186/s12913-017-2238-8

**Published:** 2017-04-19

**Authors:** Justin G. Engelbrecht, Mabjala R. Letsoalo, Admire C. Chirowodza

**Affiliations:** South to South Programme for Comprehensive Family HIV Care & Treatment, Department of Paediatrics and Child Health, Faculty of Medicine and Health Sciences, Stellenbosch, South Africa

**Keywords:** Home-based carers, Knowledge and skills, South Africa, HIV/TB services, Palliative care, Community care workers, Competencies

## Abstract

**Background:**

Home-based carers (HBCs) play a critical role in ensuring the success of the primary health care re-engineering strategy in South Africa. Their role includes ensuring improved access to and delivery of primary health care at the household level, and better co-ordination and improved linkages between community and health facilities for HIV/TB services. The objective of this study was to assess the knowledge, skills, challenges and training needs of HBCs involved in HIV/TB care in one sub-district in the North-West province of South Africa.

**Methods:**

We conducted a descriptive, cross-sectional study in which 157 HBCs were interviewed to assess their knowledge and skills regarding HIV and TB. Data were collected using a pre-tested semi-structured questionnaire. Quantitative and qualitative data were analysed using SPSS statistical software and thematic analysis respectively.

**Results:**

One hundred and forty-four (92%) of the interviewees were female and 13 (8%) were male. The median age of the participants was 35 years (interquartile range (IQR): 22–27). The median score for knowledge of both HIV and TB questions was 66% (IQR: 57–75). In general, HIV knowledge scores were higher than TB knowledge scores (73% versus 66%). A significant association was found between knowledge scores and formal training (*p* < 0.05), and knowledge scores and highest educational levels (*p* < 0.05). Irrespective of knowledge, HBCs reported providing a variety of services to support HIV/TB services in the communities in which they worked. HBCs also reported facing various challenges in their jobs related to stigma and the social contexts in which they work.

**Conclusion:**

The study showed that the overall knowledge of HBCs was limited, given the skills required and the services they provide. Given the increasing role of HBCs in various health initiatives, targeted interventions are required to support and improve their competencies and service provision.

## Background

South Africa has the highest number of people living with HIV/AIDS in the world, as well as the highest rate of tuberculosis (TB) incidence and the highest rate of drug-resistant TB in Africa [1, 2]. The huge burden of HIV/AIDS on the public health sector has prompted the government to re-engineer the health system by focusing on community outreach services and the formalisation of national community health worker (CHW) programmes. These programmes have the potential to contribute to reducing the huge human resources gap and to extend primary health-care services to communities in rural areas that are difficult to reach [3].

The importance of non-clinical health cadres, such as home-based carers, is pivotal in ensuring the success of re-engineering health care and ensuring that health facility and community linkages are strengthened. HBCs perform various health tasks in the community, such as health education and promotion of health behaviours, provision of counselling and mobilisation of communities. They also provide bereavement counselling and even assist with transporting patients to health facilities [4]. In some settings, HBCs are expected to provide daily basic care and support to HIV/AIDS and TB patients. South Africa has a national government policy to promote home-based care [5]. Most programmes rely on volunteers, who sometimes are paid small stipends for transport to affected communities to carry out basic nursing and other caregiving activities in patients’ homes [6]. These volunteers constitute a great proportion of caregivers and experience physical, emotional and socio-economic burdens that are major barriers to providing adequate services, as well as to their professional and personal development [7]. The role of HBCs in South Africa is central to the success of interventions aimed at reducing the burden on HIV/TB services. Their function in palliative care ensures clinical benefit and improved adherence to drugs, offers care to the patients, and is now an integral part of HIV/AIDS and TB management [8].

While their critical role has been acknowledged in the health setting, HBCs face many challenges. These range from burnout and impoverishment to injury, increased vulnerability to illness and emotional despair [4, 9]. Furthermore, HBCs receive no formal training that provides a systematic framework containing an appropriate curriculum for the duties and responsibilities of their jobs [10, 11]. The South African National Department of Health has also highlighted that caregivers experience emotional and physical problems, such as rejection, anger, grief, physical strain and stress, when providing care to patients confined to their beds. The community context of their work also means that they are unaware of how to tackle social issues affecting the patient, such as disclosure, adherence problems, and poor support from family and relatives [10, 11].

There is a need to assess and continuously update the competencies required to provide home-based care in the ever-changing HIV/TB landscape in South Africa. The home-based care guidelines stipulate comprehensive knowledge requirements for HBCs, including the basic definitions of HIV and the different modes of transmission [12]. HBCs need to be able to differentiate HIV from AIDS and TB, understand issues surrounding opportunistic infections and basic nutrition for an HIV-positive person, as well as provide psychosocial support [13].

South Africa has one of the greatest HIV/TB coinfection burdens on the African continent, and this has a significant impact on public health efforts [14]. According to the Annual Performance Plan 2014/15 to 2016/17 of the South African National Department of Health, TB case detection rates currently stand at 69%, compared to the global target of 70%, but there are still many missed opportunities to identify and treat existing cases at community level. The cure rate in the North West province improved from 66.4% in 2010 to 68.9% in 2011 [15]. In this context, HBCs serve communities with both a high prevalence of HIV infection as well as a high prevalence of TB. There is a high occupational risk for HBCs to contract TB, and there also is a need for HBCs to be competent in protecting themselves from acquiring TB by using personal protective equipment, which in many cases is not available. There currently is very little research on the factors that promote good TB infection-control measures amongst health-care workers (including HBCs) [16].

A cross-sectional study assessing the implementation of a community health worker programme in rural Eastern Cape, South Africa found that there was limited accredited training, as well as uneven non-formal training of this cadre of health-care providers [17].

A 2011 South African government audit established that there were more than 72 000 facility- and community-based lay health workers linked to health departments across the country. The current re-engineering model envisages a reorganisation of this community-based care infrastructure into primary health care (PHC) outreach teams of community health workers (CHWs) led by professional nurses [18]. The North West province was an early adopter of this new strategy. In 2011/12, a total of 5 167 community-based workers linked to the North West Provincial Department of Health provided home-based care and directly observed treatment, short-course (DOTS) for TB, 80% of whom had no accredited training [19]. A few studies have been conducted in South Africa assessing home-based care programmes; only two have been done in the North West province, exploring HBCs’ experiences and educational needs. Currently, all the HBCs included in this study still operate under their original names, and only a few have partnered with the re-engineering teams in this sub-district [20].

The purpose of this study was to determine whether HBCs affiliated to health-care facilities in the Moretele Sub-district in the North West province of South Africa have the appropriate knowledge, skills and expertise to provide DOTS support for TB and HIV support services. The study further aimed to report on the challenges, training gaps and services offered by the home-based care organisations that were selected as the sample in our study.

## Methods

The study was conducted in the Moretele Sub-district of Bojanala District, North West province, South Africa. Study site selection was done as part of the South to South HIV Programme implementing its District Support Model at different health-care facilities. Gaps were identified by the researchers in HBCs rendering HIV/TB care services to the local community.

The North West province is one of the smaller and more rural South African provinces. It has a population of 3.6 million, an infrastructure of 22 hospitals and 300 clinics/community health centres, and a well-established district health system consisting of four health districts and 19 sub-districts. Moretele Sub-district has a diverse population of 180 000, spread over 1 369 km^2^, and consists of rural, informal settlements and villages.

We conducted an observational, cross-sectional study to assess the knowledge, skills and attitudes of HBCs working in this sub-district. A questionnaire was developed by the researchers based on their current research experiences and knowledge of HIV and TB. The population consisted of 11 home-based care organisations affiliated with two community health care centres, making a total sample size of 157 HBCs. A purposive convenience sampling approach was used to identify individual HBCs from these organisations.

The questionnaire was piloted with an independent home-based care organisation in Gauteng province to test feasibility and validity. The study questionnaire covered a range of topics, including educational level, home-based care knowledge, formal training, and various challenges experienced by HBCs working in the context of HIV/AIDS and TB, as well as the services provided compared to their knowledge and training. The questionnaire was translated from English into Setswana to ensure standardised interviews in the participants’ home language. Data were collected and verified over a period of two months, from June to July 2012, using semi-structured interviews conducted at the HBCs organisations’ offices. Responses were documented by research assistants on the questionnaire, and interviews lasted 30 to 45 min. Informed consent was sought prior to conducting the interviews. The completed questionnaires were captured in a Microsoft Access 2007 worksheet, and then SPSS was used for data analysis. Descriptive statistics were used to understand the data collected on the knowledge, training needs and support needed by the HBCs. Ethical clearance was provided by the Health Research Ethics Committee of Stellenbosch University and the North West Health Ethics Committee. The Strengthening the Reporting of Observational Studies in Epidemiology (STROBE) guidelines have been adhered to in this study.

## Results

### Profile of HBCs sampled

A total of 157 surveys were conducted with HBCs across 11 organisations working in the Moretele Sub-district in the province. One hundred and forty-four (91.7%) of the survey participants were female, while 13 (8.3%) were male. The median age was 35 years (interquartile range, IQR: 22 to 27), with more than half (51%) of the responses being in the age group 35 years and older. Approximately 132 (84%) were fully employed, 20 (12.7%) were part-time employed, and five (3.2%) were self-employed. Approximately 138 (88%) HBCs stated that they had received stipends. Most HBCs (94; 59.9%) had a high school educational level (Grades 11 to 12), 41 (26.1%) had a secondary school education (Grades 8 to 10), while only two (1.3%) had a tertiary education.

### HIV/TB knowledge

The participants answered 12 questions regarding HIV/AIDS, TB knowledge, skills policies and guidelines. Table 1 shows the median scores of TB knowledge and HIV knowledge, and the combined total score against demographic and social characteristics. The overall knowledge of the HBCs with regard to TB and HIV care was 65%. Scores for HIV knowledge were generally higher than scores for TB knowledge (73% vs 66%). The interquartile range (IQR) includes the middle (median) of the sample scores for both TB and HIV. There was a statistical difference for the HIV/TB total knowledge score by highest educational level, as well as receiving formal training (*p* < 0.05). There was no difference for the total knowledge by age.

**Table 1:** Median scores of TB and HIV knowledge and the combined total score against demographic and social characteristics (*n* = 157)

Characteristics	TB knowledge score		HIV knowledge score (%)		Total knowledge score (HIV/TB combined)			*P* value
	Median score	IQR	Median score	IQR	Median score	IQR	Total (N)	
Age								0.751
20–24	57	46–68	80	73–80	62	57–73	28	
25–29	61	45–73	73	60–80	63	54–76	24	
30–34	71	50–87	73	53–80	69	57–84	25	
35+	66	51–77	60	53–80	66	56–73	80	
Highest educational level								
Primary	71	53–83	53	48–60	66	55–73	20	0.032^a^
High school	65	46–78	80	60–80	68	58–77	94	
Secondary	60	39–72	60	53–80	64	51–70	43	
Received formal training								
Yes	71	56–80	73	60–80	70	60–76	75	0.004^b^
No	56	42–71	73	58–80	61	53–73	82	
Overall score	66	57–75	73	60–80	66	57–75	157	

*P* values are for total knowledge score only; ^a^ Independent Samples Kruskall Wallis Test for comparison across groups; ^b^ Mann-Whitney Test for comparison between groups

*IQR* Interquartile range

Table 2 summarises the correct answers to the 12 knowledge scores exploring HIV/TB knowledge in the study. The HBCs performed exceptionally well (75% and above) for questions related to understanding the relationships between HIV and TB, as well as knowledge of the duration of TB treatment for the first episode (mean score of 96%), knowledge of impact of HIV on the body’s ability to fight disease (mean score of 94%), signs and symptoms of TB (mean score of 76%), and knowledge of signs and symptoms of AIDS (mean score of 75%, SD). The HBCs also performed above average (mean score of 60% and above) for questions related to knowledge of TB drugs (mean score of 63%), knowledge of TB treatment duration for the second period (mean score of 63%), definition of the term AIDS (mean score of 63%), and knowledge of the side effects of TB drugs (mean score of 69%). Questions that scored low marks (mean score below 50%) regarded understanding the body organs affected by TB (mean score of 37%), naming the side effects of TB drugs (mean score of 34%), and the definition of the term HIV (mean score of 14%).

**Table 2:** Means scores for correct answers to 12 questions exploring HIV/TB knowledge, policies and guidelines

	Mean % (standard deviation)
1. Understanding body organs affected by TB^b^	37 (23)
2. Signs and symptoms of TB^b^	76 (27)
3. Knowledge of TB drugs^c^	63 (48)
4. Knowledge of side effects of TB drugs^a^	69 (46)
5. Naming of side effect of TB drugs^b^	34 (30)
6. Knowledge of TB treatment duration for 1^st^ episode^b^	96 (19)
7. Knowledge of TB treatment duration for 2^nd^ episode^b^	63 (48)
8. Definition of the term HIV^c^	14 (34)
9. Definition of the term AIDS^c^	63 (48)
10. Knowledge of signs and symptoms of AIDS^c^	75 (31)
11. Knowledge of impact of HIV on body’s ability to fight disease^a^	94 (22)
12. Relationship between HIV and TB^a^	96 (18)

^a^ Binary questions, yes, no option; ^b^ three or more options provided in answer option; ^c^ open-ended question option

### Services offered by HBCs to communities

The services offered by the HBCs were mainly the provision of nutritional, psychosocial and medical care, and holistic and adherence support. HBCs reported providing a variety of services to support HIV/TB services in the communities in which they worked; they provided TB DOTS (94%), anti-retroviral therapy (ART) medication (57%), and adherence support to ART (72%), while 85% provided adherence support to TB patients. A large proportion (85%) reported that they provided nutritional support, which was often a challenge, especially when patients taking TB medication or ART cannot feed themselves. Approximately 92, 95 and 97% of the sample said that they had provided sexually transmitted, opportunistic infection and opportunistic infection prevention services to their clients respectively. Treatment for opportunistic infections was provided by 96.1% of the HBCs. A large proportion had provided emotional (99.3%) and spiritual support (92.3%).

The percentage of HBCs supporting more than three patients on TB treatment was 27% at the time of the survey, while 49.7% supported no TB patients. The percentage of HBCs supporting three or more patients on ART was 17.8%, while 54.8% mentioned that they had not supported this group at all. Of the 157, 39.4% and 50% offered social services, such as the provision of blankets, soap and consumables, and assistance with funeral arrangements respectively. Services such as how to obtain condoms (99.3%) and the provision of emotional support (99.3%) were offered by nearly all HBCs.

### Challenges experienced by HBCs in rendering services

The researchers attempted to understand the challenges experienced by HBCs in home-based care programmes aiming at promoting community and social contexts, in order to enable them to effectively care for people living with HIV/AIDS and TB in this district. Within these programmes, challenges were also reported regarding HIV/AIDS and TB services delivery, and thematic analysis was done based on the responses.

The HBCs reported various key challenges they experienced when providing services in communities. The top three frequently mentioned challenges voiced during the surveys were: confronting negative attitudes and behaviour by community members during their work (71%), difficulties in supporting the treatment of defaulters (53%), and confronting the poverty of the communities they were supporting (25.6%). Other less commonly mentioned challenging experiences included being confronted with patients with suicidal thoughts, patients needing assistance with cooking, and denial and stigma amongst community members with regard to HIV/AIDS issues.

### Feedback on training gaps

**Fig. 1:**
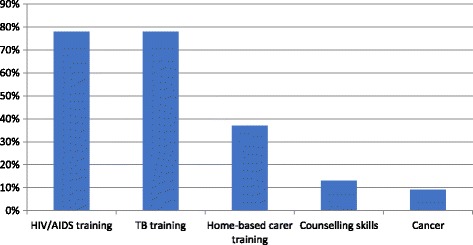
Summary of training needs as reported by HBCs

During the survey, HBCs were given an opportunity to list their training needs and topics on which they would like to undergo training. The researchers were not trained to gather information regarding the context surrounding these topics. Figure 1 provides a summary of the training requested by the HBCs during the interviews. Most HBCs regarded HIV/TB (78.3%), TB (77.7%), home-based care (36.9%), counselling skills (13%) and cancer (9%) as the top training needs. Other training needs that were not so prominent (mentioned less than five times) were monitoring and evaluation, computer literacy, first aid training, gardening, self-defence training, and financial management. Mental health training also did not feature prominently in the feedback.

## Discussion

The overall level of knowledge of the HBCs was 65%; however, only 47% indicated that they had any formal training. Approximately 60% of the HBCs had a high school educational level. Most HBCs were able to provide a comprehensive care plan to their clients, including health promotion, education, basic care and preventative care. Priority training needs included counselling skills and home-based care and HIV/TB training. Our results also highlight some key knowledge gaps of HBCs supporting critical HIV/TB services in South Africa. Specific knowledge scores for HIV were generally higher than the scores for TB, while the definitions of terms were generally poor.

Our sample included all home-based care organisations from a specific community. Our results could be generalisable to other rural, resource-limited settings in other provinces in the country. The South to South Programme for Comprehensive Family HIV Care & Treatment was supporting several health-care facilities at the time through its experienced and skilled technical advisors, who were also part of the research team.

Our findings reveal that there was a significant correlation between the formal training and educational levels of the HBCs and their overall knowledge. The availability and range of training opportunities have also been closely associated with sustained motivation among HBCs, as well as with their ability to respond appropriately to the clients’ needs [21]. Furthermore, equipping HBCs with adequate skills to address their clients’ needs has been particularly problematic in programmes that lack the capacity to provide ongoing training. The findings of a descriptive study done in the Odi Sub-district in the North West province recommended that informal caregivers and families should undergo training in order to empower them to reduce the overflow of patients in hospitals [22]. Another study, done in the same province to assess the educational and supportive needs of informal caregivers, showed that these needs were concentrated most on health promotion and disease prevention [23]. The challenges faced by the HBCs in our study were similar to the findings of a study done in semi-rural communities located in KwaZulu-Natal. These included dealing with patients’ attitudes and behaviour, poverty, treatment defaulters, stigma and denial of HIV status [7]. HBCs often are inadequately trained to cope with the mental health problems of their clients, including those relating to alcohol abuse and depression; similar results were found in a programme in Kenya [24, 25]. Formal training has also been shown to improve competencies [26].

Establishing linkages to existing programmes and services may help provide a holistic, comprehensive approach to caring for families and individuals infected and affected by HIV and TB. These linkages could include collaboration for referral and case management with social and early childhood development programmes, social work, social services, social security and food aid and health programmes [7]. Mental health practitioners should assist volunteers and carers with continuing psychosocial support in order for them to cope with their daily activities in the community. Other studies have highlighted the demands arising from home-based care, such as community members’ bad attitudes and behaviour, and confronting poverty, stigma and discrimination, which make their roles stressful and difficult [7].

The study was conducted among HBCs affiliated to two community health centres in a sub-district at a specific point in time. The sample may not have been representative of all the HBCs in this sub-district, and therefore lacks external validity. The study was able to show an association between knowledge scores and educational levels/formal training variables. These variables may only hold among HBCs under specific conditions or contexts. Further studies should therefore be conducted to include a larger sample from other health-care facilities within the sub-district. The Moretele Sub-district management should address all the challenges, training and service gaps and ensure that the current home-based care organisations are incorporated into the PHC re-engineering teams.

There is also a need to provide on-going training of HBCs, supplemented with refresher courses. Training organisations in home-based care settings should evaluate the curriculum of caregivers to ensure that the content is updated with current information [10]. Our results highlight the enormous competence development and training needs of HBCs, who play a critical role in supporting the HIV/AIDS and TB epidemic in the South African health system.

Our study also demonstrates the need to support and fast track processes for the professionalisation of HBCs in the South African context. Irrespective of the critical role they play in strengthening health systems, their dedication and passion are unlikely to be sustainable in the absence of focused, competence-based training, remuneration and recognition. Furthermore, proper professional alignment will allow the proper documentation of home-based care activities, thereby improving true reflection of health system performance for district health systems. Strengthening and institutionalising the professionalisation of HBCs will assist in the achievement of various international and national initiatives, such as primary health care re-engineering and the 90-90-90 UNAIDS global initiative, by strengthening community and facility linkages [27].

## Conclusion

This study has shown that the overall knowledge of HBCs is limited, given their required skills and the services they provide. HBCs reported the type of training they required to perform their roles, including training in HIV/TB as well as more customised TB training. There is a need for standardised training of and targeted, on-going support interventions for HBCs in order to ensure their competence to support the skills they require and services they provide in the important role they play. As the North West was an early adopter of the PHC strategy, these results may further confirm the legitimacy of the current ward-based outreach teams (WBOTs).

## Abbreviations

AIDS: Acquired Immune Deficiency Syndrome
CHWs: Community health workers
DHIS: District Health Information System
DOTS: Directly Observed Treatment, Short-Course
HBCs: Home-based carers
HIV: Human Immunodeficiency Virus
NDOH: National Department of Health
PHC: Primary health care
TB: Tuberculosis
WBOTs: Ward-based Outreach Teams
